# Characteristics of Extended-Spectrum β-Lactamase Producing Enterobacterales Isolated from Dogs and Cats, 2011–2021

**DOI:** 10.3390/vetsci10030178

**Published:** 2023-02-22

**Authors:** Dennis J. Woerde, Krystle L. Reagan, Barbara A. Byrne, Bart C. Weimer, Steven E. Epstein, Cory Schlesener, Bihua C. Huang, Jane E. Sykes

**Affiliations:** 1 William R Pritchard Veterinary Medical Teaching Hospital, University of California-Davis, 1 Garrod Drive, Davis, CA 95616, USA; 2 Department of Veterinary Medicine and Epidemiology, University of California-Davis, 1 Garrod Drive, Davis, CA 95616, USA; 3 Department of Veterinary Pathology, Microbiology & Immunology, University of California-Davis, 1 Garrod Drive, Davis, CA 95616, USA; 4 Department of Population Health and Reproduction, 100K Pathogen Genome Project, School of Veterinary Medicine, University of California-Davis, 1 Garrod Drive, Davis, CA 95616, USA; 5 Department of Veterinary Surgical and Radiological Sciences, University of California-Davis, 1 Garrod Drive, Davis, CA 95616, USA

**Keywords:** *Escherichia coli*, multidrug resistance, virulence, Enterobacterales

## Abstract

**Simple Summary:**

Emerging antimicrobial resistance is a major concern in both human and veterinary medicine. Of particular concern is the emergence of extended-spectrum beta-lactamase (ESBL)-producing bacteria. The ESBLs are a group of enzymes produced by bacteria that inactivate commonly used antimicrobials. Infections caused by ESBL-producing bacteria are increasingly being recognized in human medicine; however, information is lacking regarding the characteristics of ESBL-producing bacterial infections associated with clinical illness in dogs and cats. This study examined ESBL-producing bacterial infections in dogs and cats presenting to a veterinary teaching hospital from 2011–2021. *Escherichia coli* was the most commonly identified bacterial species, with urinary tract infection being the most common clinical presentation. Multi-drug resistance was present in 90% of ESBL-producing bacterial infections. Based on susceptibility patterns, antimicrobials such as piperacillin-tazobactam, amikacin, and cefoxitin may be alternative antibiotics to the current recommended regimen. Whole genome sequencing of bacteria was performed, which revealed *bla*_CTX-M-15_ was the most common ESBL gene identified.

**Abstract:**

The rising prevalence of extended-spectrum β-lactamase (ESBL)-producing Enterobacterales is a significant threat to animal and human health. This study aims to describe the clinical features, antimicrobial susceptibility patterns, and genotypic features of infections associated with ESBL-producing Enterobacterales in dogs and cats seen at a tertiary referral veterinary teaching hospital. Enterobacterales isolated from dogs and cats that underwent ESBL testing during the study period were identified using a search of the hospital antimicrobial susceptibility test software database. Medical records of confirmed ESBL isolates were reviewed, and the source of infection, clinical findings, and antimicrobial susceptibility were recorded. Genomic DNA from bacterial isolates was evaluated for antimicrobial resistance genes with whole genome sequencing. Thirty ESBL-producing isolates were identified based on phenotypic testing (twenty-nine from dogs, one from a cat); twenty-six were *Escherichia coli* and the remainder were *Klebsiella* spp. Bacterial cystitis was the most commonly identified (8/30, 27%) clinical problem associated with infection. Resistance to three or more antimicrobial classes was identified in 90% (27/30) of isolates, and all isolates were susceptible to imipenem. Over 70% of isolates were susceptible to piperacillin-tazobactam, amikacin, and cefoxitin. *Bla*_CTX-M-15_ was the most common ESBL gene identified, present in 13/22 (59%) isolate genomes. A wide range of clinical infections were identified. Piperacillin-tazobactam and amikacin may be alternatives to carbapenem therapy. Further, larger-scale studies are needed.

## 1. Introduction

The increasing prevalence of antimicrobial-resistant bacterial infections is of significant concern in human and veterinary medicine. One of the most important causes of antimicrobial resistance within the family Enterobacterales (previously Enterobacteriaceae) is the acquisition of plasmid-mediated production of β-lactamase enzymes, resulting in resistance to β-lactam antimicrobials. More than 500 distinct β-lactamases have been identified [1] and several classification systems have been developed [2].

Extended-spectrum β-lactamases (ESBLs) are a group of β-lactamases capable of hydrolyzing third-generation cephalosporins yet are inhibited by clavulanic acid [3]. This inhibition by clavulanic acid differentiates ESBLs from AmpC type β-lactamases, another group of β-lactamases that confer resistance to third-generation cephalosporins and monobactams [4].

The major ESBL genes associated with bacteria isolated from human and animal patients belong to the groups *bla*_CTX-M_, *bla*_TEM_, and *bla*_SHV_ [2]. These resistance genes are most prevalent in *Escherichia coli* and *Klebsiella pneumoniae*, but can also be found in *Enterobacter* spp., *Salmonella* spp., *Proteus mirabilis*, and *Pseudomonas aeruginosa* [5]. The prevalence of infections associated with ESBL-producing bacteria is increasing worldwide [6], with multidrug resistance (MDR) being a common feature [5]. This feature often leads to treatment failure with empiric antimicrobial therapy, can limit clinical therapeutic options, and in human patients can lead to an increased mortality rate compared to infections with non-ESBL-producing Enterobacterales [7]. Hence, ESBL-producing bacteria pose a serious threat to human and animal health.

A paucity of information exists in veterinary medicine regarding the prevalence of infections with ESBL-producing bacteria, their clinical characteristics, bacterial species involved, and associated resistance mechanisms. Urinary tract infections, hepatobiliary infections, respiratory tract infections, bacteremia, and intra-abdominal infections have all been reported as secondary to ESBL-producing Enterobacterales in human medicine [8]. Several studies have identified ESBL-producing bacteria in the feces of healthy cats and dogs [9,10]; however, information is lacking regarding the phenotypic and genetic characteristics of isolates associated with clinical illness in dogs and cats.

The aims of this study were to describe the clinical features, antimicrobial susceptibility patterns, and genotypic features of ESBL-producing Enterobacterales infections in dogs and cats presenting to a veterinary teaching hospital. 

## 2. Materials and Methods

### 2.1. Isolate Identification, Antimicrobial Susceptibility Test Results & Patient Data

Enterobacterales isolates that underwent ESBL testing from July 2011 to July 2021 were identified using a search of the database of the antimicrobial susceptibility testing software (Sensititre SWIN, V3,2,3 and V3.3, ThermoFisher Scientific, Waltham, MA, USA) used by the University of California-Davis Veterinary Medical Teaching Hospital Veterinary Microbiology laboratory. Briefly, a subset of the *Escherichia coli* and *Klebsiella* spp. isolates from dogs and cats that were identified as resistant to cefpodoxime (range of concentrations tested, 0.25–32 µg/mL) using broth microdilution (Sensititre, ThermoFisher Scientific COMP1F, COMPAN2F, or COMPGN1F panels) were further tested for ESBLs using the ESBLF panel (Sensititre, ThermoFisher Scientific).

Isolates that had at least a three, two-fold dilution difference in minimum inhibitory concentration (MIC) between one or both of cefotaxime (range of concentrations tested, 0.25–32 µg/mL) or ceftazidime (range of concentrations tested, 0.25–128 µg/mL) and when these antimicrobials were combined with clavulanic acid were classified as ESBL-producing isolates [11]. Quality control strains tested with all panels included *E. coli* American Type Cell Collection (ATCC), *Staphylococcus aureus* ATCC 25923, and *Pseudomonas aeruginosa* ATCC 27853. Susceptibility results were compiled and reported for each antimicrobial tested. Bacterial isolates were classified as susceptible, intermediate, or resistant using current veterinary-specific CLSI breakpoints [12] available at the time of writing. Urinary-specific breakpoints were used for isolates obtained from the urinary tract. Where veterinary-specific breakpoints were not available, human breakpoints were used. Multidrug resistance (MDR) was defined as resistance to three or more classes of antimicrobials [13]. All susceptibility testing was done at the time of isolation.

From 2014 to 2021, bacteria were identified to the species level using matrix-assisted laser-desorption ionization time-of-flight mass spectrometry (MALDI-TOF MS; Bruker, Billerica, MA, USA), and spot oxidase and indole testing. Prior to 2014, Enterobacterales were identified using conventional biochemical testing, including triple sugar iron, citrate, Christensen’s urea agar (Hardy Diagnostics, Santa Maria, CA, USA), and sulfur-indole-motility agar (Biological Media Service, University of California, Davis, CA, USA), the aforementioned spot tests, or identification strips (API 20E, BioMerieux, Durham, NC, USA).

Identified bacterial isolates were then cross-referenced to individual patient medical records through a search of the University of California-Davis Veterinary Medical Teaching Hospital database (VMACS; Veterinary Medical and Administrative Computer System, University of California-Davis) to obtain data on signalment, clinical features, and specimen source.

### 2.2. Whole Genome Sequencing

Whole genome sequencing (WGS) of available banked isolates was performed. Banked isolates were stored as frozen stabilates at −80 °C using a bead-based system (Pro-Lab Microbank ^TM^, ThermoFisher Scientific) until sequencing. Isolates were revived by subculture from the frozen stabilates on 5% sheep blood agar (Hardy Diagnostics, Santa Maria, CA, USA) and incubated overnight at 35 °C in 5% CO_2_.

WGS was performed using the methods from the 100K Pathogen Genome Project [14,15]. Briefly, high-molecular-weight genomic DNA (gDNA) was extracted from banked bacterial isolate colonies using the Wizard Genomic DNA Jit (Promega, Madison, WI; cat#A1460) as described previously [16].

Approximately 600 ng of purified genomic DNA was used to construct a sequencing library using the KAPA HyperPlus library preparation kit (Roche Diagnostics). Library size distribution verification was performed on the Caliper LabChip GX (Perkin Elmer), and library quantification was performed with the KAPA Library Quantification Kit (Roche Diagnostics). Pooled libraries were sequenced on the Illumina HiSeq X Ten using the PE150 protocol. Reads were trimmed with Trimmomatic [17], assembled with SPAdes [18], and annotated with prokka [19], all with default settings. Antibiotic resistance genes were analyzed in every isolate genome using the Comprehensive Antibiotic Resistance Database (CARD) [20].

### 2.3. Genomic Similarity Comparison

Multi-Locus Sequence Typing (MLST) calls were made by scanning genome assemblies using “mlst” software version 2.23.0 [21] using PubMLST typing schemes [22]. Genome Assemblies were compared by Jaccard similarity index of k-mer (size = 31 bases) sequence-based profiles (sketch size = scaled 100 k/Mbp) using Sourmash version 3.2.3 [23]. Sequences were uploaded to the Sequence Read Archive database [24].

## 3. Results

### 3.1. Isolate Identification, Antimicrobial Susceptibility Test Results & Patient Data

**Figure 1:**
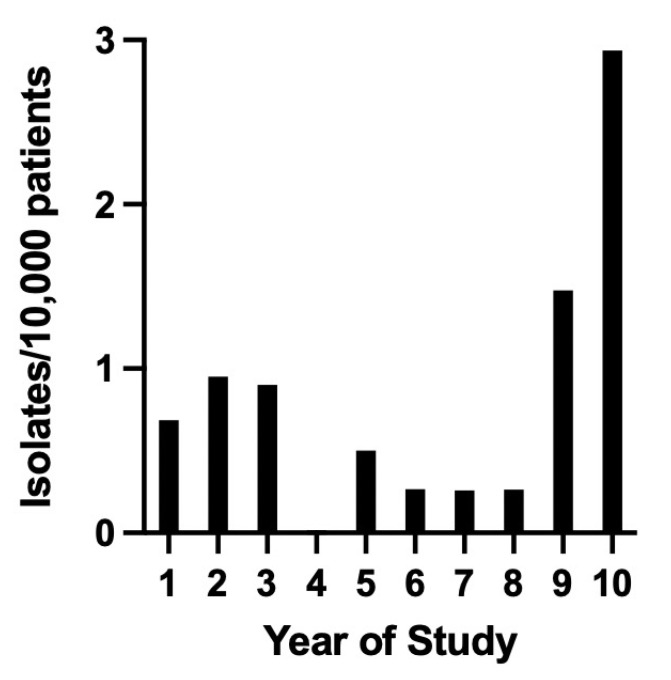
Prevalence of ESBL-producing Enterobacterales (isolates/10,000 patients for each year of the study, year 1 being July 2011–June 2012).

A total of 30 ESBL-producing bacterial isolates were identified among approximately 5300 susceptibility panels performed over the 10-year study period on *Escherichia coli* and *Klebsiella* spp., 22 of which had been banked. Twenty-nine of the isolates were from dogs, and one was from a cat. A total of 299,187 dogs and 55,698 cats were examined at the hospital over the same 10-year period. Dogs were 5.3 times more commonly seen than cats at the hospital in the 10-year period of the study. The number of ESBL-producing Enterobacterales isolated varied per year (Figure 1); however, more than half (17/30) were obtained from the final two years of the study. Of the 30 isolates, 26 were *Escherichia coli*, 3 were *Klebsiella pneumoniae*, and 1 was *Klebsiella oxytoca*.

**Figure 2:**
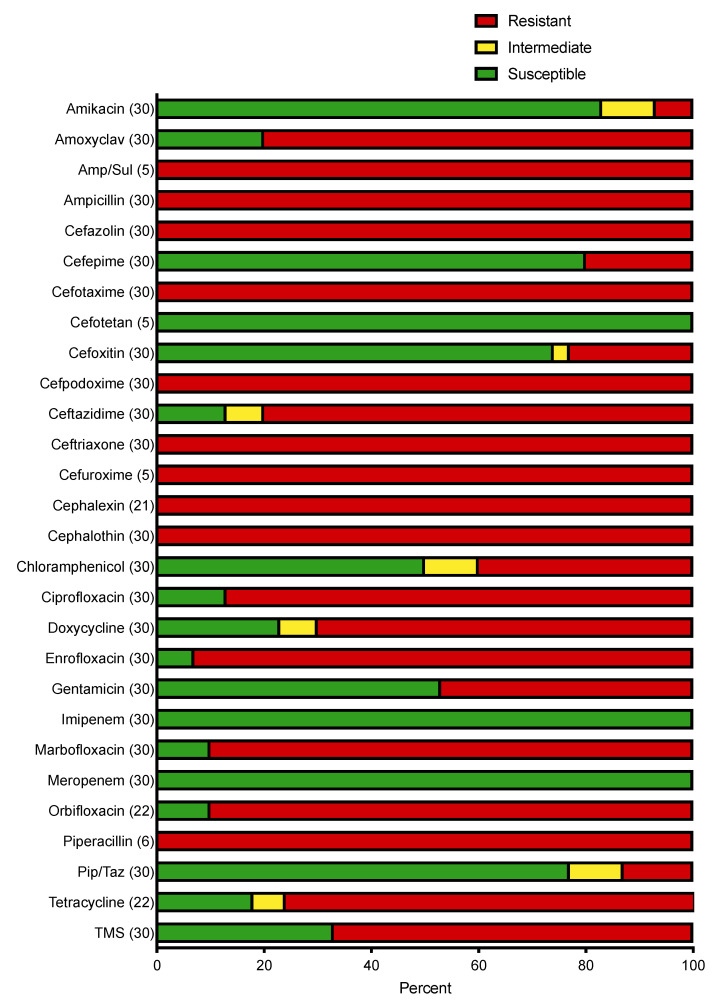
Susceptibility patterns for ESBL-producing bacterial isolates identified expressed as a percentage (susceptible, intermediate, and resistant, as defined by CLSI breakpoints; antimicrobials for which breakpoints were not available are not included). Number of isolates tested against each antimicrobial is listed on the vertical axis in parentheses. Amp/Sul, ampicillin/sulbactam; Amoxyclav, amoxicillin/clavulanic acid; Pip/Taz, piperacillin/tazobactam; TMS, trimethoprim sulfamethoxazole.

Antimicrobial susceptibility results of the isolates are summarized in Figure 2. All isolates were resistant to aztreonam, ampicillin/sulbactam, and piperacillin. The majority of isolates were resistant to the fluoroquinolones, including enrofloxacin (93%), marbofloxacin (90%), and orbifloxacin (90%). All isolates were susceptible to imipenem, meropenem, and cefotetan. Over 70% of isolates were susceptible to piperacillin-tazobactam, amikacin, and cefoxitin (Figure 2). MDR was identified in 27/30 (90%) of isolates. Resistance to cefepime, a 4th generation cephalosporin, was identified in 6/30 (20%) isolates.

Bacterial isolates were cultured from urine obtained via cystocentesis (11/30), skin swab specimens (7/30), pleural effusion collected via thoracocentesis (2/30), liver fine needle aspirate specimens (2/30), blood (2/30), peritoneal effusion collected by abdominocentesis (2/30), and one each of bronchoalveolar lavage (BAL) fluid, cholecystocentesis bile fluid, transtracheal lavage (TTL) fluid, and an ear swab specimen. Of the 11 bacterial isolates from urine, 8 were from dogs that had lower urinary tract signs consistent with bacterial cystitis (pollakiuria, hematuria, or malodorous urine). The remaining 3 isolates were from dogs without signs of lower urinary tract disease; 1 dog had clinical features consistent with pyelonephritis, and the other 2 dogs had subclinical bacteriuria but were being treated with glucocorticoids, and there was concern that this treatment may have been masking clinical signs of lower urinary tract disease. Two ESBLs were cultured at different time points from the same dog; an ESBL-producing *Klebsiella pneumoniae* was identified on the initial urine culture, and a urine culture performed 4 weeks later revealed the same ESBL-producing *K. pneumoniae* as well as an ESBL-producing *E. coli*.

Of the 4 isolates from the respiratory tract, 3 were from dogs that had clinical signs and radiographic abnormalities that were consistent with aspiration pneumonia. The remaining isolate was from a dog with pyothorax secondary to migrating grass awn. The cat with an ESBL-producing *E. coli* infection had bacterial peritonitis secondary to a dog bite wound.

### 3.2. Whole Genome Sequencing

A total of 22/30 isolates were available for whole-genome sequencing, 4 *Klebsiella* spp. and 18 *E. coli* isolates, all from dogs. Genes associated with ESBL production were identified in each genome (Table 1). *Bla*_CTX-M-15_ was the most common ESBL gene identified (13/22 isolate genomes). All isolates had multiple genes contributing to beta-lactam resistance. Either AmpC or AmpH β-lactamase genes or both were detected in all 22 isolates.

### 3.3. Genomic Similarity Comparison

**Figure 3:**
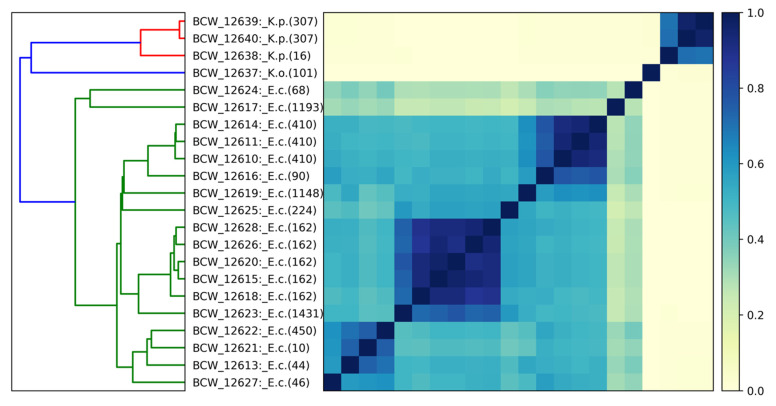
Sample genome assembly comparisons (DNA sequence k-mer (31 bp) matching) are shown in all against all matrix by Jaccard similarity index (color scale on right: 1.0 (dark blue) is near identical, 0.0 is no observed relationship). A hierarchical clustering dendrogram (left) shows structure of relatedness (branch lengths represent relative distance between clusters, colors correspond to species). Samples are labeled by Sequence Read Archive (SRA) ID number, species (E.c.: *Escherichia coli*, K.p.: *Klebsiella pneumoniae*, K.o.: *Klebsiella oxytoca*), and PubMLST schemes (*Escherichia coli* [Achtman], *Klebsiella pneumoniae* species complex [Pasteur], and *Klebsiella oxytoca*), respectively. The plot shows clustering corresponding to species and MLST.

Examination of sample identity by multi-locus sequence typing (MLST) [22] and comparison of genomic distance were carried out (Figure 3). Clustering of genomes was observed, but this did not correlate with the sample collection site (material, body location). Clusters with high genetic similarity were observed among isolates with the same MLST number, with the largest groups being *E. coli* (162) (n = 5, modest genetic diversity) and *E. coli* (410) (n = 3, modest genetic diversity). Two *E. coli* isolates (BCW_12624 and BCW_12617) showed greater divergence from each other and the remainder of the examined *E. coli* samples. Therefore, the *E. coli* isolates examined were diverse, and 12 MLST groups were identified.

## 4. Discussion

ESBL-producing Enterobacterales were associated with a wide range of clinical presentations in this study. Most of the specimens in this study in which ESBL-producing Enterobacterales were detected were from the urinary tract. Given that *E. coli* is the most common pathogen associated with urinary tract infections in dogs [25], this result was expected. The prevalence of ESBL-producing bacterial isolates in human patients with bacteriuria has been as high as 37% in some studies [26].

Almost all the ESBL-producing Enterobacterales in the study reported here were from dogs, with only one isolate from a cat. Furthermore, it is possible the isolate from the cat originated from the oral flora of a dog, given the source was dog-bite-associated peritonitis. A 2021 meta-analysis of studies pertaining to ESBL-producing *E. coli* in dogs and cats isolated from a variety of specimens however revealed no difference in the global prevalence of ESBL-producing Enterobacterales between dogs and cats [27]. Our hospital examined a total of 5.3 times more dogs than cats over the 10-year period, which may have contributed to the higher proportion of isolates from dogs. In addition, the prevalence of urinary tract infections (and bacterial infections in general) in cats is low compared to dogs [25], likely contributing to the lower number of isolates from cats in this study. The number of ESBL-producing Enterobacterales isolated varied per year (Figure 1), and more than half (17/30) were obtained from the final two years of the study. When examining the hospital data over the 10-year period, only 28% more patients were evaluated in 2020–2021 compared to 2011–2012, so the increase could not be explained by patient volume alone. However, conclusions about a change in the prevalence of ESBL-producing Enterobacterales cannot be drawn from this study because of inconsistent testing across the study period.

Multidrug resistance was a common feature of the identified isolates, present in 90% of isolates in this study. ESBLs typically confer resistance to third-generation cephalosporins, although, by definition, fourth (cefepime) and fifth (ceftaroline) generation cephalosporins remain active against ESBL-producing bacteria [28]. In this study, 6/30 (20%) isolates were resistant to cefepime. Carbapenems are considered the treatment of choice for ESBL-producing Enterobacterales infections in humans [29], and all isolates in our study were susceptible to carbapenems. Several clinical studies in human patients have revealed higher rates of mortality in patients with infections caused by ESBL-producing Enterobacterales treated with cefepime than those treated with carbapenems [30]. However, carbapenem-resistant Enterobacterales have been identified [31], and strategies to reduce carbapenem use should be employed when feasible. In the current study, 77% of isolates were susceptible to piperacillin/tazobactam, an extended-spectrum penicillin and beta-lactamase inhibitor combination antimicrobial with a broad spectrum of activity against Gram-positive and Gram-negative bacteria. Whilst some conjecture in the literature exists between in vitro activity and clinical responses, a study in human patients with urinary tract infections caused by ESBL-producing *E. coli* revealed that carbapenem and piperacillin/tazobactam had similar clinical efficacy and mortality rates were similar between groups [32]. Similarly, 83% of isolates in this study were susceptible to amikacin, so this represents an additional alternative antimicrobial for the treatment of ESBL-producing Enterobacterales infections, at least for the population of animals seen at the authors’ institution. Isolates were largely susceptible to cefoxitin and cefotetan (second-generation cephamycins). ESBLs typically do not hydrolyze cephamycins, although isolates resistant to cephamycins have been reported [28]. Several studies of human patients have reported the efficacy of cephamycins in the treatment of urinary tract infections caused by ESBL-producing Enterobacterales [33], although data are limited on their use for the treatment of infections outside the urinary tract or in small animals. 

*Bla*_CTX-M-15_ was the most prevalent ESBL gene and is the most common ESBL gene reported in *E. coli* isolates from dogs and cats worldwide [27]. The prevalence of various ESBL genes in bacteria from companion animals may vary geographically, although the number of isolates in these studies is low. The *bla*_TEM_ gene family was the most common in a Brazilian study (47 isolates) [34], *bla*_CTX-M-1_ in a French study (10 isolates) [35], and *bla*_CTX-M-14_ in New Zealand (36 isolates) [36]. A study performed in 2011 that characterized ESBL genes from 54 *E. coli* isolates from companion animals in the USA revealed 78% of isolates carried the *bla*_CTX-M-15_ gene [37]. Concerningly, all isolates in our study possessed multiple genes that could contribute to beta-lactam resistance.

While isolates in this study were obtained from patients with clinical disease, ESBL-producing Enterobacterales have been detected in the feces and saliva of healthy dogs and cats [9,10]. Future studies are needed to determine whether there are differences in the phenotypic and genotypic characteristics of ESBL-producing bacteria from diseased versus healthy companion animals. Additional studies are also needed to identify risk factors for infection and the outcomes of infection. In human medicine, significant previous antimicrobial use [38,39], urinary catheter placement [38], and overcrowded households [39] have all been implicated as risk factors for ESBL colonization. In one study that examined the presence of ESBL-producing Enterobacterales in fecal specimens from healthy dogs in the Netherlands [10], consumption of raw meat was the main risk factor identified. A similar study performed in the UK on the fecal carriage of ESBL-producing Enterobacterales [40] found that dogs with a history of antimicrobial therapy in the past year and dogs obtained from a shelter or breeder were at increased risk for colonization. Recently, the presence of ESBL-producing Enterobacterales in the feces of dogs and cats was investigated in animals admitted to a veterinary hospital in Brazil on admission and at discharge [34]. A total of 11/47 patients had ESBL-producing Enterobacterales in their feces at hospital discharge and not at admission, suggesting veterinary hospitals as a major source of acquisition. Evidence of household transfer of ESBL/AmpC-producing Enterobacterales among humans and dogs has been reported [41], although other studies have suggested exposure to a common environmental source or clonal transmission between pets and humans is more likely [10].

There were several limitations to this study. Only 22 of the 30 isolates were banked and available for WGS. The results reflect the population of bacteria and their resistance patterns seen at the University of California-Davis, which is a tertiary referral hospital, and may not reflect bacterial populations seen at other hospitals. Given the high prevalence of AmpC and AmpH β-Lactamase genes identified in this study, some ESBL-producing Enterobacterales may have been overlooked using our search process. This is because ESBL detection is often masked by high-level production of AmpC [42]. Susceptibility testing and ESBL confirmation were not performed on all *E. coli* and *Klebsiella* isolates obtained during the study period. As a result, meaningful temporal information on prevalence may be lacking. Furthermore, routine susceptibility panels did not include all antimicrobials, such as nitrofurantoin.

## 5. Conclusions

This study lays the groundwork for future large-scale epidemiological studies on dogs and cats caused by clinical infections with ESBL-producing Enterobacterales. Improved understanding of prevalence, risk factors, outcomes, and mechanisms of resistance gene acquisition is needed for companion animals. Information on clinical cases will ideally help clinicians identify animals that are at high risk for ESBL infections and aid in antimicrobial selection. Surveillance for the emergence of ESBL-producing Enterobacterales is important in both veterinary medicine and human healthcare, given the potential for zoonotic and healthcare-associated transmission.

## Figures and Tables

**Table 1:** Beta-lactamase genes identified in each isolate after whole-genome sequencing.

Isolate	Organism	Year Isolated	Source	SRA Name	MLST Number	ESBL Gene Product	Other β-Lactamase Gene Products
1	*Escherichia coli*	2012	BAL fluid	BCW_12610	410	CTX-M-15	AmpC, AmpH, OXA-1
2	*Klebsiella pneumoniae*	2020	Urine	BCW_12638	16	CTX-M-15, SHV-1, TEM-1	AmpH, CBP-1
3	*Escherichia coli*	2021	Urine	BCW_12625	224	CTX-M-1	AmpC, AmpH
4	*Escherichia coli*	2021	Urine	BCW_12626	162	CTX-M-14, TEM-1	AmpC, AmpH
5	*Escherichia coli*	2021	Skin	BCW_12627	46	CTX-M-15	AmpC, AmpH, CMY-136
6	*Klebsiella pneumoniae*	2021	Skin	BCW_12640	307	CTX-M-15, SHV-28	AmpH, CBP-1
7	*Escherichia coli*	2020	Pleural fluid	BCW_12621	10	CTX-M-15	AmpC, AmpH
8	*Escherichia coli*	2020	Skin	BCW_12622	450	CTX-M-15	AmpC, AmpH
9	*Escherichia coli*	2017	Urine	BCW_12615	162	CTX-M-14	AmpC, AmpH
10	*Escherichia coli*	2013	Bile	BCW_12611	410	CTX-M-15	AmpC, AmpH, OXA-1
11	*Escherichia coli*	2019	Bile	BCW_12616	90	CTX-M-15, TEM-1	AmpC, AmpH
12	*Escherichia coli*	2013	Skin	BCW_12614	410	CTX-M-15, TEM-1, FONA-6	AmpC, AmpH, OXA-1
13	*Escherichia coli*	2013	Ear swab	BCW_12613	44	CTX-M-15, FONA-6	AmpC, AmpH, OXA-1, DHA-1
14	*Escherichia coli*	2019	Urine	BCW_12617	1193	CTX-M-27	AmpC, AmpH, CMY-12
15	*Escherichia coli*	2020	Blood	BCW_12620	162	CTX-M-14	AmpC, AmpH
16	*Escherichia coli*	2019	Skin	BCW_12619	1148	FONA-6, TEM-1	AmpC, AmpH
17	*Escherichia coli*	2019	Tracheal wash	BCW_12618	162	CTX-M-14, TEM-1	AmpC, AmpH
18	*Klebsiella oxytoca*	2012	Urine	BCW_12637	101	TEM-1, SHV-66	AmpH, OXY-2-10
19	*Escherichia coli*	2021	Urine	BCW_12628	162	CTX-M-14, TEM-1	AmpC, AmpH
20	*Escherichia coli*	2021	Urine	BCW_12624	68	CTX-M-15	AmpC, AmpH, OXA-1
21	*Escherichia coli*	2021	Urine	BCW_12623	1431	CTX-M-15, TEM-1	AmpC, AmpH, CMY-2
22	*Klebsiella pneumoniae*	2021	Pleural fluid	BCW_12639	307	CTX-M-15, SHV-28, TEM-1	AmpH, OXA-1, CBP-1

BAL, bronchoalveolar lavage; SRA, sequence read archive; MLST, multi-locus sequence typing; ESBL, extended-spectrum β-Lactamase.

## Data Availability

Further inquiries can be directed to the corresponding authors.
